# ‘Wild Type’

**DOI:** 10.1099/mic.0.001495

**Published:** 2024-08-30

**Authors:** Isabel Askenasy, Jemima E. V. Swain, Pok-Man Ho, Rahan Rudland Nazeer, Amelie Welch, Éva Bernadett Bényei, Leonardo Mancini, Sivan Nir, Pinyu Liao, Martin Welch

**Affiliations:** 1 Department of Biochemistry, Hopkins Building, Tennis Court Road, Cambridge, CB2 1QW, Cambridge, UK

**Keywords:** *Escherichia coli*, evolution, laboratory strain, *Pseudomonas aeruginosa*, wild-type

## Abstract

In this opinion piece, we consider the meaning of the term ‘wild type’ in the context of microbiology. This is especially pertinent in the post-genomic era, where we have a greater awareness of species diversity than ever before. Genomic heterogeneity, *in vitro* evolution/selection pressures, definition of ‘the wild’, the size and importance of the pan-genome, gene–gene interactions (epistasis), and the nature of the ‘wild-type gene’ are all discussed. We conclude that wild type is an outdated and even misleading phrase that should be gradually phased out.

## Background

One of the most widely used (and possibly abused) expressions in the modern discipline of microbiology is the term ‘wild type’. Although originally popularized in the early 20th century by Hunt Morgan and colleagues to describe the phenotype and genotype of fruit flies, the term has now become pervasive across the whole of biology. But what does ‘wild type’ actually mean, especially in the context of microbiology? This is worth revisiting, if only because almost every microbiology paper uses the term. Moreover, the meaning of ‘wild type’ has not been critically reviewed in the microbiology literature since the 1960s [1] – well before the current transformative era of genomics. Even back then, and as Demerec *et al*. noted, the wild type was somewhat arbitrarily defined [1].

Microbiology is a broad discipline and the term ‘wild type’ means different things to different people. For some, ‘wild type’ conjures up characteristics or properties associated with environmental or clinical isolates (i.e. the organism ‘in the wild’), whereas for others the term is simply used to define e.g. the parent/progenitor strain of a mutant or even just a widely used domesticated laboratory strain. For yet others, and to paraphrase Holmes, ‘wild type’ describes an individual organism or allele deemed ‘normal’ or typical for the species [2]. However, given the wealth of genomic data now available to us, we believe that the expression ‘wild type’ needs reappraisal and that there should be a transition towards more meaningful terminology. To be clear, our goal is not to say what the wild type should be, but rather to highlight the limitations of the term – particularly for those tempted to draw species-wide generalizations based on the behaviour of domesticated laboratory strains or small numbers of clinical/environmental isolates. The focus of our commentary is on bacteria, and for reasons of space limitation we have selected exemplars from just a handful of widely studied species. Nevertheless, the general arguments we raise likely apply to all microbial species.

## Some examples of wild types

First, a little bit about the background to some well-known ‘wild types’. Perhaps the most widely used wild type on the planet is *Escherichia coli* K12. Interestingly, K12 was originally isolated in 1922 from a patient recovering not from a classic enteric infection, but from diphtheria [3]. K12 is essentially avirulent and grows well on laboratory media, although after over a century of domestication (and bombardment with mutagens to cure it of its F-plasmid [4]) it could be argued that the organism has little in common with either its environmental or clinical cousins, or its progenitor [5]. K12 and its descendants also harbour a frameshift (just a single base deletion) in the *rph* operon, leading to polar effects on the downstream *pyrE* ORF and subsequent slower growth during pyrimidine limitation [6]. The ubiquitous *E. coli* DH strains, such as DH5α and DH1, are K12 derivatives engineered by Douglas Hanahan to be more transformable. For example, DH5α carries mutations in *endA1* and *recA1*, and is suitable for blue–white selection due to the *lacZ*ΔM15 deletion (which removes residues 11-41 of LacZ, rendering the product active only in the presence of the *lacZ* alpha fragment). Other widely used laboratory strains, such as BL21, are not K12 derivatives, but instead derive from *E. coli* B [7]. The pedigree of the latter is somewhat murky, although it is clear that it was isolated by Félix d’Herelle (the co-discoverer, along with Twort, of bacteriophage) at the Institut Pasteur some time around 1918 [8]. Strain B and its derivatives have greater membrane permeability than K12, and are a better chassis when it comes to studies of bacterial evolution.

A particular favourite of the current authors is *Pseudomonas aeruginosa*, which claims two popular domesticated wild types; PAO1 and UCBPP-PA14. The former is a spontaneous chloramphenicol-resistant mutant of an Australian isolate (‘PAO’), originally sourced from an infected wound by Bruce Holloway in 1954 [9]. (The chloramphenicol resistance derives from a loss-of-function mutation in *mexS*. This leads to constitutive expression of a chloramphenicol efflux pump, MexEF-OprN [10,11]). PAO1 was described as growing ‘luxuriantly’ on nutrient agar, and was shown to be genetically amenable [9]. By contrast, UCBPP-PA14, more commonly known simply as PA14, was isolated in the 1970s from a patient in Pennsylvania (USA) with a burn wound [12]. PA14 is far more virulent than PAO1, likely due (in part) to its acquisition of a mutation in *ladS* [13]. PA14 is also resistant to the antibiotic rifampicin [14]. We will come back to the significance of genetic integrity later on. Unlike PAO1, which is a representative of a minor clonal lineage (designated ‘clone W’), PA14 appears to be representative of the most common circulating *P. aeruginosa* clone worldwide (clone A) [15]. The phylogenetic tree of the species has recently been further refined based on high-resolution genomic analyses [16].

The brief overview above serves to illustrate two things. First, our existing, widely used wild types have sometimes been selected for very specific reasons, and may not be representative of the population as a whole. The *P. aeruginosa* clone A strain, PA14, is an exception to this, although somewhat incongruously, PAO1 (clone W strain) remains far more widely used as a laboratory model. Second, a common feature of many widely used laboratory wild types is that they grow rapidly, display some easily assayable phenotypes, and/or are genetically amenable (or have been engineered to be genetically amenable). These are not necessarily features typical of the species as a whole. Indeed, many field- or clinic-derived isolates are often rather slow growing and genetically rather intractable.

## The heterogeneity problem

The usual interpretation of ‘wild type’ – note that the term is hyphenated only when used as an adjective – is that it represents a fit genetic configuration of an organism in its natural environment. However, two decades’ worth of genomic analyses have revealed that strains^1^ of a given species isolated from ‘the wild’ often display considerable diversity, both in terms of genetic integrity (i.e. heterogeneity within the core genome^2^) and accessory genome content^3^ [17,18]. This is well illustrated by *P. aeruginosa*, which has a habit of colonizing diseased human airways, such as those in persons/people with cystic fibrosis (pwCF). Cross-sectional genomic analyses show that although the infecting strain in a given pwCF is usually genomically homogenous, it often undergoes a burst of adaptive radiation shortly after the initial colonization event [19,20]. Furthermore, the colonizing populations continue to evolve over time, presumably in response to ongoing challenges, such as onslaught from the immune system, aggressive antibiotic intervention, and the presence of a variety of competing microbes [21]. Moreover, even within a given pwCF, we see spatial heterogeneity too, in that different lobes of the lung support genomically distinct sub-populations [22]. Interestingly, though, and in spite of their genomic divergence, these isolates display a remarkably convergent transcriptomic programme [23]. These data throw into question the notion of a single optimally fit *genomic* configuration; rather, it is more a case of ‘many roads lead to Rome’. In the light of this, which one of the (potentially) hundreds or even thousands of different variants in a single pwCF is ‘wild type’? Choose a different patient, and you’ll have hundreds more unique derivatives to choose from. And so on.

## Where and what exactly is ‘the wild’?

This is pertinent because, for many species, it is rather difficult to define what ‘the wild’ actually is – especially for organisms that are capable of occupying a range of disparate environments. For example, and as many people find out the hard way, pathogenic variants of *Escherichia coli* are just as at home in our waterways as they are in our GI tract, yet the former environments are usually cold and rather nutrient-limited, whereas the latter are warm and nutrient-replete. It follows that selection pressures in the two environments are likely to be radically different. Furthermore, the vehicle linking transmission between these two environments might be something as innocuous as a bean sprout [24], and the ability of the organism to survive on such substrata before eventually being consumed potentially layers on additional adaptive requirements. As noted above, the co-habitant species in each environment also play a part, and given that some of these co-habitants are likely to compete with the pathogen, this introduces an additional, if poorly defined, ecological selection pressure.

There are also a number of misconceptions about ‘the wild’. For example, for decades, researchers have made statements along the lines of ‘*Pseudomonas aeruginosa* is a ubiquitous micro-organism, often found on the surfaces of plants and animals, and in soil and water’ or suchlike. However, although it can and has been isolated from all these sources, it turns out that it is actually rather hard to find outside of the built environment [25]. Consequently, some of our assumptions about microbial ecology are flawed and/or perpetuate earlier mistruths; ‘the wild’ can be a difficult thing to pin down.

## Horizontally acquired DNA

It could be argued that for pathogenic species such as *E. coli*, the initial infecting strain is closest to the wild type, but wild type for what? The roadside ditch or the human gut? And given that *E. coli* likely cycles between these different environments, is there such a thing as an ‘initial infecting strain’? Unfortunately, bacteria do not come with a logbook detailing their individual natural histories, but if they did, it would likely tell a colourful story. More often than not, the bacteria’s solution to such cyclic lifestyle changes is either to dump genetic elements and become a specialist in one or other niche, or to collect additional ‘genetic baggage’, often (but not exclusively [26]) in the form of horizontally acquired accessory genomic elements. To obtain a sense of the diversity conferred by these genomic elements, a recent study revealed that across ~1300 *P*. *aeruginosa* isolates, the core (conserved) genome comprised 665 genes, whereas the accessory ‘pangenome’ comprised >53 000 genes [27]. Similarly, an analysis of 307 *E. coli* genome sequences revealed ~780 core genes and some 23 000 pan-genes [28]. Clearly, our obsession with just a handful of wild types has come at the cost of understanding what the pan-genes do.

Horizontally acquired DNA has the potential to radically alter bacterial physiology, and there is evidence to suggest that the ‘regulatory scars’ left behind as DNA is successively acquired and then lost during the evolutionary history of an organism are not without consequences. A vivid example of this is seen in *Serratia marcescens* [29]. *Sma* 12 is a ‘wild- type’ clinical isolate, whereas *Sma* 274 is a ‘wild type’ veterinary isolate (originally obtained in 1922 from a sample of milk in the Netherlands). *Sma* 12 is non-pigmented but carries a functional quorum sensing system (i.e. is QS^+^). By contrast, *Sma* 274 is pigmented but is QS^−^. Phage-mediated transfer of the functional QS system from *Sma* 12 into *Sma* 274 led to pigment production in the latter coming under QS control. This remarkable ‘genomic memory’ of one-time QS regulation was also seen when the pigment biosynthetic cluster from *Sma* 274 was introduced into the non-pigmented but QS^+^ strain (*Sma* 12). Here, pigment production in the recipient came under control of the *Sma* 12 QS system. This is a nice example of how horizontally acquired DNA can have large-scale and highly consequential effects on gene expression.

Indeed, bacteriophage may influence physiology substantially beyond their simple capacity to act as vehicles for the transfer of genetic material. The temperate phage of *P. aeruginosa* are reported to affect quorum sensing, biofilm formation and interactions with the host immune system [30], all of which can be modulated by curing the host of its phage [31].

More generally, strain-specific accessory genomic elements provide the organism with a toolkit that it can dip into when circumstances demand. The contents of this toolkit are often highly variable, but they clearly do confer a fitness advantage [32,33]. Interestingly, individual clones of *P. aeruginosa* appear to prefer a specific repertoire of accessory elements, suggesting that the core and accessory genome segments are not randomly assembled [15]. Furthermore, in both *E. coli* and *P. aeruginosa*, epistatic interactions between core genes and horizontally acquired genomic elements are now known to radically alter gene essentiality [34,35]. This is all pertinent because another feature of many well-established domesticated wild type strains is that they carry rather little in the way of accessory genome, or have been deliberately engineered to reduce their accessory genome content to a minimum. In this regard, while they are great experimental chassis for functional analysis of the core genome, they are very unlike most ‘true’ wild types.

## Laboratory wild types are in a continual state of evolution

Even once we have isolated a strain, designated it ‘wild type’, sequenced its genome, and frozen or lyophilized aliquots for archiving and dissemination, we still face problems. Genomes are never static, and biochemical ‘slop’, as well as the grinding inevitability of chemical tautomerism (reviewed in [36]), means that lesions in DNA are a fact of life. Although most of these lesions are repaired before they become fixed as single-nucleotide polymorphisms (SNPs) or indels, some inevitably slip through the statistical net. Of course, this low probability is offset by the vast numbers of cells in a rapidly dividing culture, such that after a few hours, there will be hundreds of different variants present, even assuming that the initial inoculum was a single, genomically homogenous bacterial cell.

The unrelenting nature of mutation brings us to a third, and altogether more insidious, problem; that our supposedly well-defined, domesticated laboratory wild types are in a continual state of evolution. For example, in 2010, Klockgether *et al*. used a combination of physical mapping and next-generation sequencing to compare isolates of the common laboratory strain of *P. aeruginosa*, PAO1. Specifically, they compared the PAO1 that was used to obtain the reference genome sequence (PAO1-UW) with that of PAO1 sourced from a culture collection (PAO1-DSM) and a lineage of PAO1 that has been used to generate a widely used community resource, the University of Wisconsin two-allele transposon mutant bank (MPAO1) [37]. This analysis revealed that the lineages differ due to a plethora of SNPs, indels, large-scale gene cluster duplications, and a 2.2 Mbp inversion. Importantly, these differences had measurable impacts on the fitness, virulence, and antimicrobial resistance of each lineage. Remarkably, Klockgether *et al*. also reported that certain sublines of PAO1-UW have acquired a Pf1-like prophage (RGP42) that is not present in the reference genome [37]. This prophage can only have been acquired during domestic passaging. As noted in the previous section, the presence of prophage can potentially have a large impact on the physiology of the organism. More recently, Chandler *et al*. carried out a similar genotypic/phenotypic analysis of PAO1 lineages donated from a selection of major research laboratories across North America [38]. Their conclusion, like that of Klockgether *et al*., is that PAO1 is undergoing continual microevolution in the laboratory setting, and that this microevolution is not without functional consequences.

Dorman and Thomson came to a similar conclusion when investigating the ongoing evolution of *Vibrio cholerae* strain NCTC 30, originally isolated from a British soldier convalescing in Egypt in 1916 [39]. They note that NCTC 30 (which, interestingly, encodes a functional β-lactamase, even though the strain was isolated long before β-lactam antibiotics were introduced into the clinic) has since clearly undergone a good deal of evolution *in vitro* [40]. These authors also draw parallels with the ongoing laboratory evolution of several domesticated *E. coli* strains. A corollary of all this is that, when requesting a mutant from a collaborating laboratory, it is probably a good idea to also request the corresponding wild type from which it was derived and/or sequence the genome of both before proceeding.

## *In vitro* selection pressures

Another corollary of ongoing laboratory microevolution is that most well-established wild-type strains are now adapted to growth in common laboratory media such as lysogeny broth (LB). (In this regard, we now know that progression of *E. coli* K-12 through the exponential part of the growth curve in LB is far more complex than previously thought [41]). However, to capture physiologically relevant phenotypes, more exotic media are often required, such as artificial wound medium [42] or artificial sputum medium (ASM) [43]. Interestingly, we found that when a PAO1 derivative was introduced into ASM, many genes displayed a pronounced signature – based on their *d*_N_/*d*_S_ value – of negative selection *cf*. growth in LB [44]. The most likely reason for this is that after decades of passaging in LB, PAO1 has become thoroughly adapted to this medium, such that a change in medium composition imposes a strong selection pressure. Consequently, we recommend prolonged passaging of wild types in any new medium prior to extensive further experimentation.

## What is a ‘wild-type gene’?

The discussion above leads us to our fourth, somewhat more philosophical, point, and that is, ‘how do we define a wild-type gene?’ Even in the absence of a selection pressure, genes drift and a lineup of the amino acid sequences of any given open reading frame (ORF) will likely reveal a good deal of intra-species variation. But which of these variants (alleles) is wild type? Furthermore, not all genes are expressed in laboratory growth conditions; presumably, and based on the principle of use-it-or-lose-it, such genes may be susceptible to degradation. As noted above, this may be particularly relevant for domesticated lineages, which are generally maintained in a rather narrow set of growth conditions. By way of example, when the transcriptome and proteome of *E. coli* REL606 (an *E. coli* strain B derivative) was assessed across 34 different commonly used laboratory growth conditions, no evidence could be found for expression of 186 of the 4379 genes encoded [45]. Presumably, this subset of non-expressed genes will be subject (in the long term) to progressive loss of function in laboratory growth conditions.

## Genetic context and epistasis

To further confuse the situation, a common assumption is that most ORFs in a given domesticated wild type are functional, although this is not always the case. Indeed, some important phenotypic characteristics associated with widely used wild types are determined (in part) by loss-of-function mutations, e.g. *ladS* and PA14 [13] or *luxS* in DH5α [46]. Moreover, and pertinent to the discussion above about the genome integrity of wild-type strains within and between different laboratories, lineages of PAO1 also display a marked tendency towards acquiring loss-of-function mutations in *mexT* [10,47]. This is important, and not only because *mexT* is an important regulator in its own right; it also has proven epistatic interactions with other global regulatory genes, such as the quorum sensing master regulator, *lasR* [48,50]. (In this regard, it is worth noting that both genes represent mutational hotspots in the clinic and in the laboratory [47,51, 52], and similar hotspotting of mutations in domesticated laboratory lineages has also been reported for other species [40].) Indeed, such gene epistatic interactions are likely a very widespread phenomenon (and can be very useful when untangling mechanistic interactions [53]), although we are only just beginning to scratch the surface of the problem.

## Closing comments

We hope that the discussion above serves to highlight the inadequacy of the term ‘wild type’ in microbiology. Our current domesticated strains have served (and are serving) us well in terms of understanding the basic biology of organisms – remaining useful backgrounds for comparison against isogenic mutants and, ideally, permitting inter-laboratory reproducibility. However, whole-genome sequencing and the introduction of massively orthogonal investigative tools are forcing us to re-evaluate just how much these models actually represent the species as a whole [34].

We suggest that the expression ‘wild type’ is now so redundant – and indeed even misleading in the context of many studies – that its use in many circumstances should be actively discouraged, for all the reasons outlined above. Given the proven laboratory-to-laboratory variation in reference lineages, we further suggest that more care is taken in defining *exactly* which progenitor strain is being used in a given study. For example, if the MW team makes a mutant in, say, PAO1, that PAO1 should be reported in the accompanying publication as PAO1_MW_ (subscript indicating that this is the PAO1 lineage routinely used in the Welch laboratory). If we receive a mutant from Another Laboratory, the experimental reference strain for that would be PAO1_AL_. And so on. This approach is not only good scientific practice in terms of pedigree tracing; it also reinforces the notion that an observed phenotype is relative to the named/defined progenitor, without making any presumption about the contribution of that phenotype to survival or growth in ‘the wild’.

We are not commenting here on *which* experimental reference strains should be used, although efforts to identify potentially more suitable ones are ongoing (e.g. [54]). In this regard, Fontana *et al*. have been implementing multi-omics-based approaches to define the optimal reference strains representing gut bifidobacterial species [55], and there is no reason why similar approaches should not be applied to other organisms. We also note that advances on this front will likely need to be accompanied by the parallel development of a genetic toolbox for each designated reference.

A second recommendation is that whichever strains are selected for downstream study, their genome should be sequenced on a moderately regular basis (and the sequence made publicly available), and care should be taken to mimimise passaging of primary stocks on laboratory media. It would probably be a good idea to sequence the genome of any newly received experimental reference strain upon receipt, and best practice to report any changes *cf*. the sequence obtained by the provider in subsequent publications. That would certainly make pedigree records easier to construct and curate.

## Footnotes

We have tried to avoid entering into the controversial taxonomic world that aims to define strains, clones and species, although these terms have been discussed by Dijkshoorn *et al.* in their very readable 2000 article [56].(17. Dijkshoorn L, Ursing BM, Ursing JB. Strain, clone and species: comments on three basic concepts of bacteriology. 2000;49(5):397-401.)

Genes that are common between most lineages of the species. The core genome is usually described as being subject to purifying selection, leading to conservation.

Genes, usually associated with horizontally- acquired genomic islands, that likely confer an advantage in specific environmental scenarios.

## Abbreviations

pwCF: person with cystic fibrosis
